# Aging Behavior and Precipitates Analysis of Wrought Al-Si-Mg Alloy

**DOI:** 10.3390/ma15228194

**Published:** 2022-11-18

**Authors:** Fang Liu, Fuxiao Yu, Dazhi Zhao

**Affiliations:** School of Materials Science and Engineering, Northeastern University, Shenyang 110819, China

**Keywords:** wrought Al-Si-Mg alloy, artificial aging, transmission electron microscopy, precipitates, double aging peaks

## Abstract

Aging behavior of wrought Al-12.7Si-0.7 Mg alloy was investigated during isothermal aging at 180 °C. Two aging peaks were observed at 3 h and 8 h, respectively. To examine precipitate evolution during aging, the alloy’s microstructure in different aging states was investigated by regular and high-resolution transmission electron microscopy (TEM and HRTEM). The results revealed that the variation of mechanical properties is attributed to the combining effect of Si particles, the grain boundary, and the character of precipitates. The predominant precipitates’ type, size, and volume fraction vary as aging time increases.

## 1. Introduction

Both cast Al-Si-Mg and Al-Mg-Si alloys, containing Mg and Si as the primary alloying elements, are strengthened by precipitation of the metastable precursors of the Mg_2_Si phase. The generally accepted precipitation sequence of Al-Mg-Si alloys is: supersaturated solid solution→Guinier–Preston (GP) zones→β′′→β′→β [1,2,3]. The mechanical strength of these alloys originates mainly from the metastable phase β′′ which has the largest hardening effect for this type of material [4,5,6]. The crystal structure of the β’’ (Mg_5_Si_6_) phase has been identified as a C-centered monoclinic structure by using HRTEM observations, ab initio calculations, and atom probe tomography (APT) [7,8,9]. However, several kinds of random-type precipitates have been reported as a transition phase between the GP zone and the β′′ precipitate [4,5]. Initial-β′′ and pre-β′′, which are in the very early monoclinic stage of β′′, have been identified experimentally with compositions close to Mg_2_Si_2.6_Al_6.4_ and (Mg_4_Si_1_) Al_6_, respectively [5]. Sha et al. confirmed that the β′′ phase contains many Al atoms by using APT studies [10]. Most aging precipitation studies have been focused on wrought Al-Mg-Si alloys. A short overview of all these phases is given in Table 1.

In terms of cast Al-Si-Mg alloys, most investigations mainly focus on the effects of solution treatment, artificial aging, Si content, the secondary dendrite arm spacing (SDAS), and alloying elements on the mechanical properties of this class of alloys without analyzing metastable precursors of the Mg_2_Si phase [11,12,13,14]. This is related to their microstructure of hypoeutectic and eutectic Al-Si alloys, which are composed of α-Al and a eutectic mixture of Al-Si in the inter-dendritic region. In this study, the aging behavior and precipitates analysis of extruded Al-Si-Mg alloy was investigated. The microstructure of deformed Al-Si-Mg alloy consists of Si particles distributed on the fine Al grain boundaries produced by the combination of direct chill casting and plastic deformation [15], and that is different from cast Al-Si-Mg alloys with dendrites and eutectic Si.

This work reports that double aging peaks are present in the aging hardening curves of the Al-Si-Mg alloy. However, Li et al. [16] reported that double aging peaks phenomena were found in cast Al-Si-Cu-Mg cast alloy aged at a temperature above 175 °C, while not in the Al-Si-Mg alloys. To analyze the reason for the variation of properties, the present research investigates the thermal stabilities of the precipitates of wrought Al-12.7Si-0.7 Mg formed during isothermal aging (180 °C) for various times.

**Table 1:** Overview of the crystal structures of metastable phases in the Al-Mg-Si system.

Metastable Phase	Crystals Structure and (or) Lattice Parameters	Chemical Compositions	Grow Direction/Orientation Relationships with Matrix
GP-zone [4]	unknown	Mg/Si~1	-
initial-β′′ [5]	Monoclinic	Mg_2_Si_2.6_Al_6.4_	<100>_Al_-oriented needles
pre-β′′ [5]	Monoclinic	(Mg_4_Si_1_) Al_6_	<100>_Al_-oriented needles
pre-β′′ [4]	Monoclinic	(Mg +Al)_5_Si_6_	-
β′′ [7,17,18]	Monoclinic, *C*2/m, a = 1.516 ± 0.002 nm, b = 0.405 nm, c = 0.674 ± 0.002 nm, β = 105.3 ± 0.5°	Mg_5_Si_6_	<100>_Al_-oriented needles
β′′ [8]	Monoclinic	Mg_5_Si_6_	[130]_Al_//[100]_β′′_,[001]_Al_//[010]_β′′_, [$\bar{3}$20]_Al_//[001]β′′
β′ [19]	Hexagonal, P$\bar{6}$2 m, a = 0.71 nm, c = 0.405 nm	Mg/Si ≈ 1.39	fully coherent with the <001>_Al_ along the c-axis
β [20]	FCC, a = 0.635 nm	Mg2Si	-
B′ [21]	Hexagonal, a = 1.04 nm, c = 0.405 nm	Mg/Si ∼ 1	-
B′ (also called type C) [3]	Hexagonal, a = 1.04 nm, c = 0.405 nm	-	(001)_B′_//(001)_Al_, [2$\bar{1}\bar{1}$0]_B′ ∧_ [100]_Al_ = 10°

## 2. Materials and Methods

An Al-Si-Mg alloy with Si 12.7 wt.%, Mg 0.7 wt.%, Fe 0.2 wt.%, Al Bal. was prepared by direct chill (DC) casting and preheated at 470 °C for 4 h before extrusion. The extrusion alloy was solution heat treated at 520 °C for 30 min in a salt bath and subsequently quenched into water at room temperature. Then, artificial aging was carried out immediately at 180 °C in a Muffle furnace for up to 12 h.

The specimens for the tensile test were cut from the extrusion profiles along the longitudinal direction. All the tensile tests were performed at a constant speed of 1 mm/min using a SANS testing machine. The mechanical data for each condition were the average from the measurements of three specimens. Elongation was measured as the engineering strain at fracture.

The samples for optical observation were prepared following standard metallographic methods. An optical microscope (LeicaDMI 5000 M, Leica Microsystems GmbH, Wetzlar, Germany) was used for this purpose. Regular and high-resolution transmission electron microscopy (TEM and HRTEM) were performed. The TEM samples were thinned to 50 μm by mechanical polishing and punched into 3 mm disks before the experiments. The samples were prepared by twin jet electropolishing with a 30% HNO_3_ and 70% Methanol solution at −20 °C and observed with an FEI Tecnai F30 (Thermo fisher Scientific, Waltham, MA, USA) transmission electron microscope operated at 200 kV.

## 3. Results

**Figure 1:**
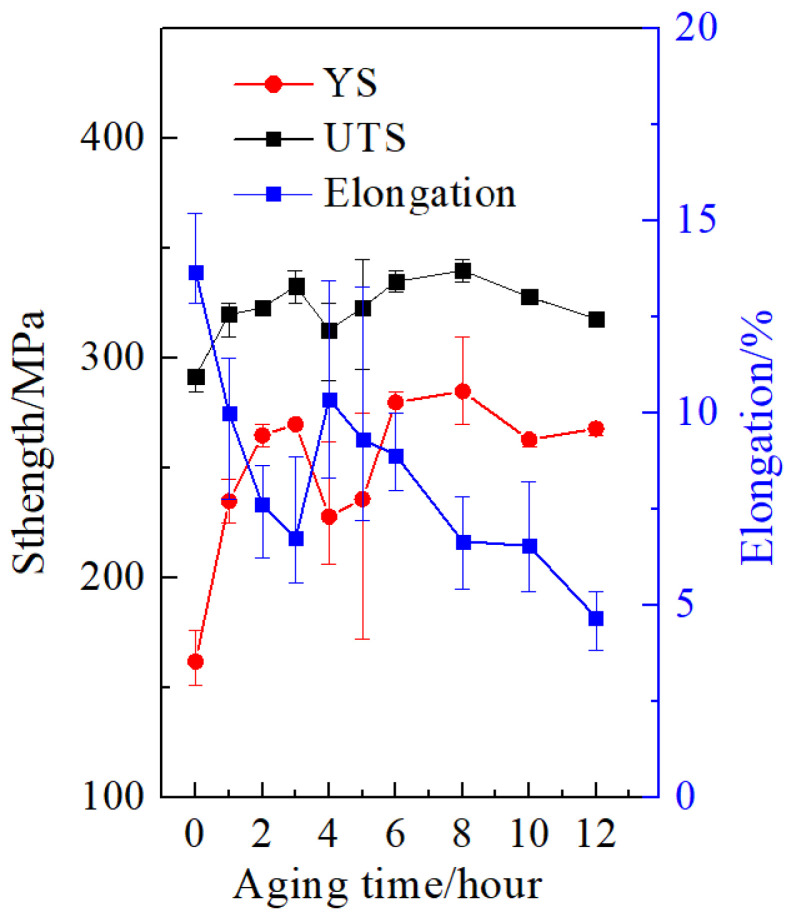
The mechanical properties of the alloy as a function of aging time at 180 °C.

The mechanical properties of the alloy as a function of aging time at 180 °C were plotted in Figure 1. The mechanical properties of the alloy present a continuous and pronounced increase in strength with increasing aging time from 1 to 3 h. It can be seen that the first peak (peak I) occurs at 3 h. The yield strength (YS) and ultimate tensile strength (UTS) of the first peak are 270 MPa and 333 MPa, respectively. After artificial aging for 4 h, the YS and UTS significantly decreased to 228 MPa and 313 MPa, respectively. It can be observed that the YS and UTS show a lower value than the alloy aged at other times. With prolonged aging time, the strength of the alloy shows an increasing trend at times longer than 5 to 8 h, and then the strength of the alloy decreases again. The second peak (peak II) appears at 8 h, and the YS and UTS are 285 MPa and 340 MPa, respectively. The strength of the second peak is slightly larger than that observed at the first peak. In addition, the change of elongation has an opposite trend before aging for 8 h.

**Figure 2:**
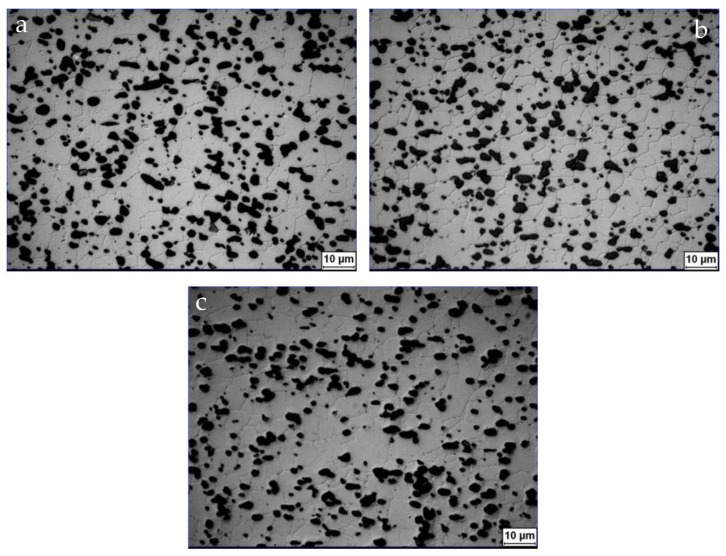
Optical micrographs of as-extruded alloy after heat treatment: (**a**) 520 °C × 30 min + 180 °C × 1 h, (**b**) 520 °C × 30 min + 180 °C × 2 h, (**c**) 520 °C × 30 min + 180 °C × 12 h.

Figure 2 is the optical microstructure of the alloy after artificial aging for 1, 3, and 12 h. It consists of black Si particles distributed uniformly in the Al matrix of fine equiaxed grain according to the analysis in ref. [15]. It can be seen that no significant changes were observed in the optical microstructure as the microstructural variations during aging are primarily the formation and evolution of precipitates.

**Figure 3:**
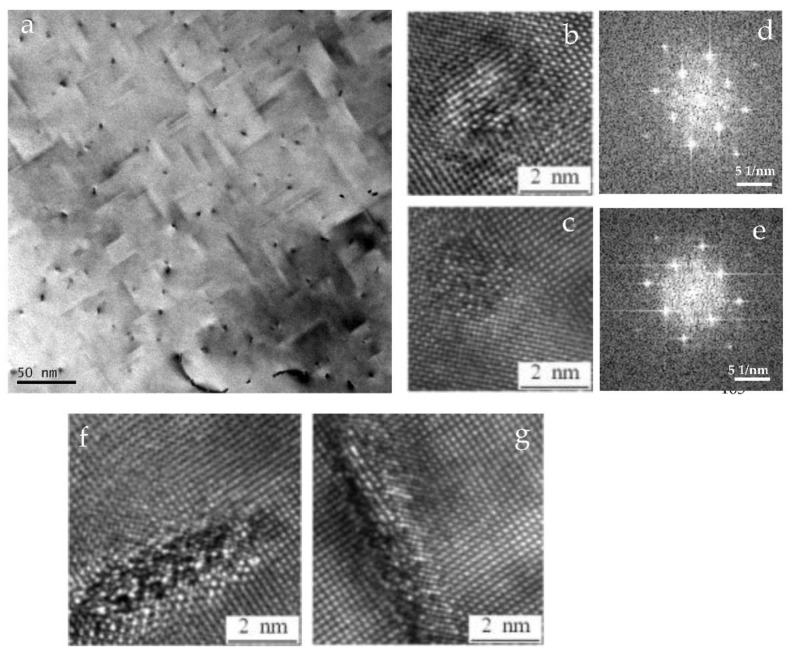
(**a**) Bright-field TEM images of the as-solutionized alloy aged at 180 °C for 1 h with zone axis [001]_Al_, (**b**–**e**) the HRTEM images of the cross-section of precipitates and the corresponding FFT of the HRTEM images, (**f**,**g**) the HRTEM images of the B’ precipitate.

Figure 3 consists of TEM bright-field images (B = [001]_Al_) from the alloy aged for 1 h at 180 °C. The needle-shaped precipitates are oriented along [010]_Al_ and [100]_Al_ directions and are visible together with some dark dots. The latter is the cross-section of needles along [001]_Al_ and appears, therefore, point-like in the viewing direction. As shown in Figure 3a, a high density of needle, very fine precipitate, and dislocations co-exist in the matrix. The needle-shaped precipitates have a mean length of about 30 nm. HRTEM images of the cross-section of needle-like precipitates with a diameter of about 2 nm are presented in Figure 3b,c which are the predominant precipitates after aging for 1 h. The shape of the cross-section of the precipitates is generally either nearly circular or circular with one or more bulges. The Fast Fourier Transformation (FFT) of the HRTEM image of the phase in the matrix is faint and diffuse, as shown in Figure 3d,e. Accordingly, the diffuse streaks are uncertain of the crystal structure of the precipitates. According to the characteristics of FFT and HRTEM, it can be confirmed that these precipitates are pre-β′′ phase, which is consistent with the analysis in ref. [5]. Figure 3f,g show the HRTEM micrograph of a lath-shaped precipitate. The angle between the long face of the precipitate cross-section and the [100]_Al_ ([010]_Al_) is about 10 degrees in Figure 3f. The morphology and orientation are very close to the structure of the B′ precipitate that was reported by Dumolt et al. [21] and Edwards et al. [1]; therefore, these precipitates in Figure 3f,g are B′, which accounts for merely a lesser proportion.

**Figure 4:**
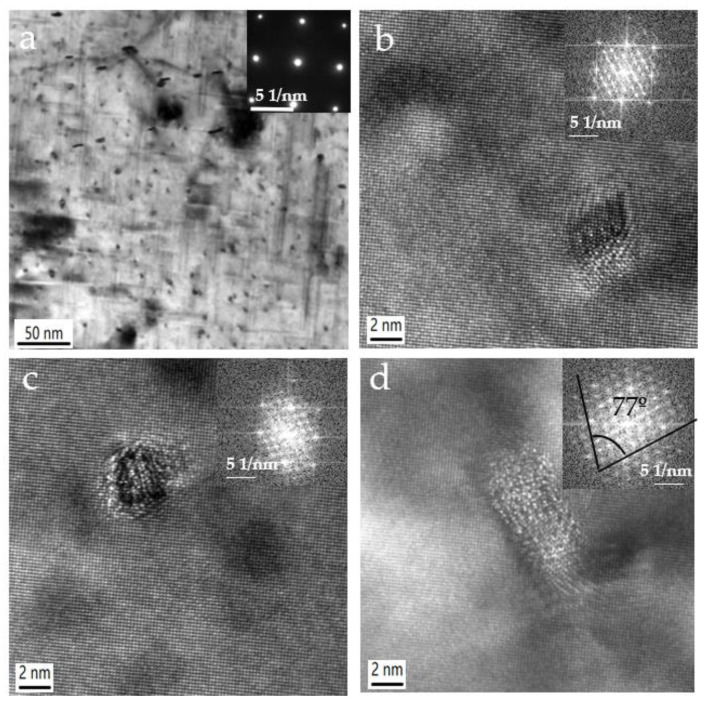
(**a**) Bright-field TEM images of the as-solutionized alloy aged at 180 °C for 3 h with zone axis [001]_Al_, (**b**–**d**) the HRTEM images of a cross-section of precipitates and the Fast Fourier transform of the HRTEM image.

Figure 4 shows the bright-field TEM image (B = [001]_Al_) of the alloy aged at 180 °C for 3 h. The length of the needle-shaped and the diameter of the cross-section precipitates increase when the aging time increased. The needle-shaped precipitates homogeneously distribute in the matrix and have a mean length of about 100 nm. In Figure 4b–d, HRTEM images illustrate the structure of precipitates, and the FFT is embedded in the corner of images. The shape of these precipitates is a parallelogram and nearly circular in cross-section view with [001]_Al_ matrix direction. Based on the HRTEM observations, we specified the following three types of precipitates (Figure 4b–d). The percentage of these types of precipitates in Figure 4b (2 × 4 nm) and Figure 4c (2 × 3 nm) is high, while the proportion of the precipitate in Figure 4d (3.5 × 7 nm) is minimal. The precipitates in Figure 4b,c are still pre-β′′, and the shape and size of the precipitates have changed compared with the precipitates in Figure 3. The lattice fringes intersect angle in Figure 4c is 77°. The appearance of the precipitates and the streaks on the FFT agrees with previous observations for β′′ precipitates [7,18]. It indicates that a small number of pre-β′′ phases have transformed into β′′ as increasing aging time to 3 h.

**Figure 5:**
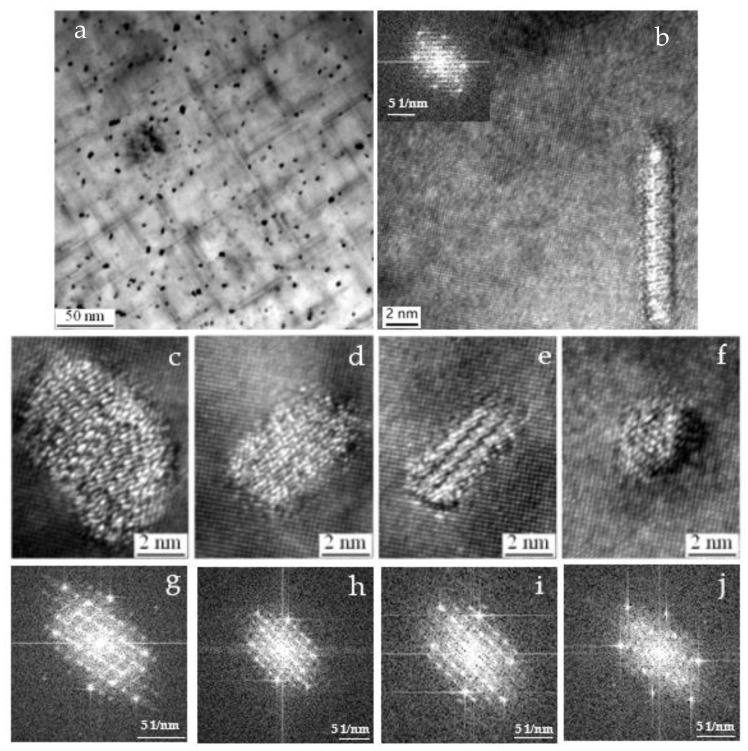
(**a**) Bright-field TEM images of the as-solutionized alloy aged at 180 °C for 4 h with zone axis [001]_Al_, (**b**–**f**) the HRTEM images of a cross-section of precipitates, (**g**–**j**) the Fourier transform of the HRTEM images in (**c**–**f**), respectively.

Figure 5 shows the bright-field TEM (B = [001]_Al_) and HRTEM images from the specimen aged for 4 h at 180 °C. As can be seen from the picture, both the length and the area of the cross-section of needle-shape precipitates increase, which leads to the YS and UTS decreasing. Dark dots appeared more clearly on the matrix, which is different from what is shown in Figure 4. Fast Fourier Transformation of images in Figure 5g indicated that the crystal structure is β′′, and the lattice fringes intersect angle in Figure 5g is about 75°. The volume fraction of large-size precipitates in Figure 5c increases and the volume fraction of small precipitates in Figure 5e,f decreases. This showed that more pre-β′′ have transformed into β′′ after aging for 4 h. Thus, the predominant precipitate phase at this aging state is pre-β′′ and β′′. B′ precipitate phase also exists in the matrix, as shown in Figure 5b.

**Figure 6:**
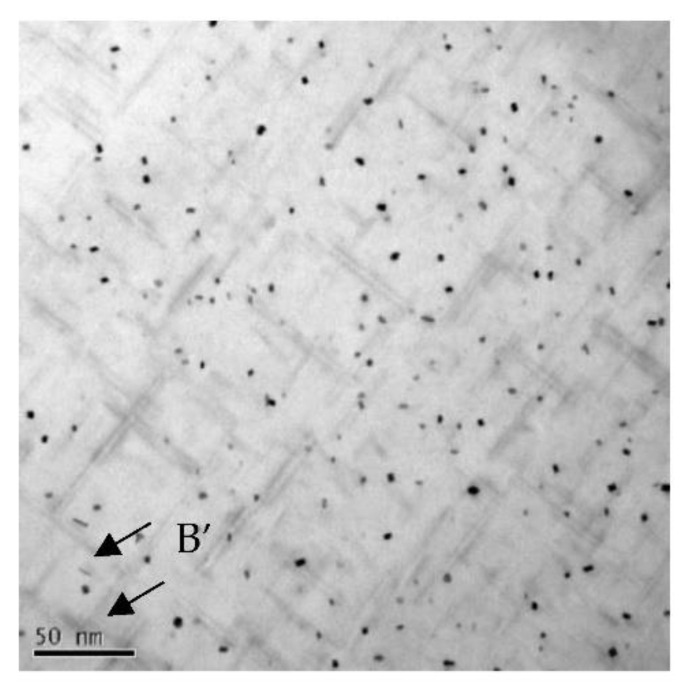
Bright-field TEM images of the as-solutionized alloy aged at 180 °C for 8 h with zone axis [001]_Al_.

**Figure 7:**
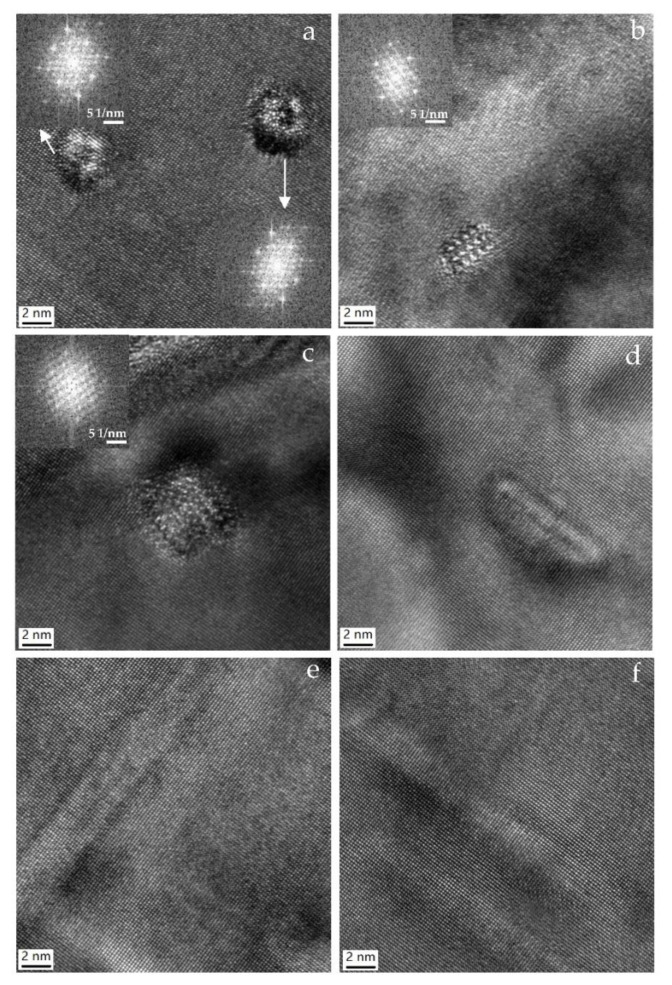
HRTEM images of the precipitates aged at 180 °C for 8 h and the fast Fourier transform of the HRTEM image. (**a**–**f**) HRTEM images of precipitates and the FFT of the HRTEM images.

Figure 6 shows a bright-field TEM micrograph (B = [001]_Al_) from a specimen aged 8 h at 180 °C. The HRTEM images of the precipitates and the Fast Fourier transform of the HRTEM images are shown in Figure 7. The length of the needle-shaped precipitate and the area of the cross-section decrease. There is a short needle-shaped precipitate shown in Figure 7d. The HRTEM images of needle-shaped precipitates oriented along [010]_Al_ and [100]_Al_ directions are shown in Figure 7e,f. They are generally lapping over in the matrix and coherent with the matrix. The B′ precipitate phase (arrowed in Figure 6) also can be observed. Fast Fourier Transformation of images in Figure 7a–c indicated that the crystal structure is monoclinic. The lattice fringes intersect angles in Figure 7a–c are from 75° to 78°. These indicated that the predominant precipitate phase after aging for 8 h at 180 °C is β′′ phase and metastable phase pre-β′′ has transformed into β′′ phase.

**Figure 8:**
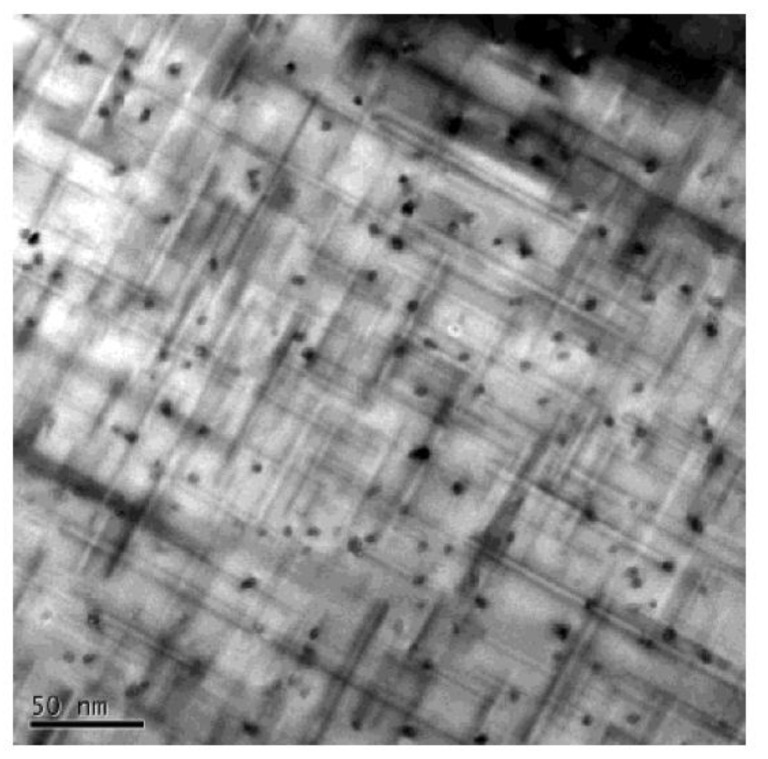
Bright-field TEM images of the as-solutionized alloy aged at 180 °C for 12 h with zone axis [001]_Al_.

Figure 8 is a TEM bright-field micrograph from a specimen aged for 12 h at 180 °C. Both the length and the volume fraction of precipitate increased distinctly compared with the alloy aged for 8 h. The average length of precipitates increased from 63 nm to 165 nm. During the increased aging time from 8 to 12 h, β′′ phase grows steadily, and the strength declines.

## 4. Discussion

There are no notable differences in the optical microstructure in Figure 2 when varying the aging time at 180 °C. The microstructure consists of fine equiaxed grains of Al matrix with uniformly distributed fine Si on the grain boundaries. Based on the observation of precipitates by TEM and HRTEM, three kinds of strengthening precipitates were being precipitated, including pre-β′′, β′′, and a little of B’. The pre-β′′ phase was first formed after aging for 1 h. Further aging to 3 h led to an increase in the size of pre-β′′ and the partial transformation from pre-β′′ to β′′. As aging proceeded, the pre-β′′ phase grew larger, and more metastable β′′ began to form in situ on the pre-β′′ phase and grew by consuming the pre-β′′ phase [10]. The precipitation sequence is in good agreement with previous results [4,5]. Therefore, the strengthening mechanism of the alloy was attributed to the combining effect of Si particles, the grain boundary, and precipitation strengthening.

The important influence of the aging time on the properties of the aged alloy should be addressed by the type, size, and volume fraction of precipitate [3,22]. The average length of the needles varies as follows: 1 h: 30 nm, 3 h: 100 nm, 4 h: 200 nm, 8 h: 63 nm, and 12 h: 165 nm. The cross-section area of the precipitate aged for 1 h is under 4 nm^2^. It varies from 4.2 to 5.8 nm^2^ when the sample aged for 3 h and continues to increase after aging for 4 h, when most of the area is around 7.5 nm^2^. As the precipitates form and grow, the strength increases gradually and reaches the first peak at 3 h. Due to the length and the area cross-section of the precipitates continuously increasing after aging for 4 h, the mechanical properties decrease. On the other hand, the atomic matching, or coherency, between the lattices of precipitates and the matrix affect the mechanical properties as well. The predominant precipitates are pre-β′′ precipitates after aging for 1 h, which is coherent with the matrix. The coherent pre-β′′ produces an increased strain field in the matrix and a further increase in strength compared with the solid solution state. With further aging, the pre-β′′ precipitates grow longer and some β′′ is formed from the transition lattice pre-β′′ simultaneously. Continuing to increase the aging time to 8 h, nearly all pre-β′′ precipitates have transformed into β′′, and the volume fraction and length of precipitates decrease; however, the strength of this aged alloy is higher because the structural phase transition toward a more stable β′′ structure starts to take place and β′′ has better hardening effect than pre-β′′ [4,5,6]. As the length is increasing for β′′ precipitates after aging for 12 h, the YS and UTS decrease. The featured stage of the evolving precipitate agrees with the alloy’s mechanical properties. Precipitates with smaller sizes and higher amounts benefit precipitation strengthening [3,10]. The large-size precipitates in Figure 5 (aging for 4 h) and Figure 8 (aging for 12 h) increase, resulting in decreasing YS and UTS.

The hardening precipitates processing is a rather complex evolution in Al-Si-Mg and Al-Mg-Si alloys, which involves changes in composition, structure, and morphology [5]. Recently, a very early monoclinic stage (initial-β′′) has been identified experimentally with a composition close to Mg_2_Si_3_Al_6_, and some Mg sites in the pre-β′′ and β′′ also contain Al atoms [4,5]. The content of Al in the structure of the precipitates increases with the degree of coherency in the Al matrix [4]. Therefore, the structure of precipitates in Figure 3b,c are more similar to the Al matrix than that of the β′′ phase. Marioara et al. [4] reported that the evolution of the hardness for 6082 Al-Mg-Si alloy aged at 150 °C for a time varying from 4 h to 43 days exhibits the double aging peaks shape of the hardness curve due to the existence of two different phases pre-β′′ and β′′. Chen et al. showed that precipitates dissolve by the continuous replacement of Al atoms with Mg and Si in the precipitates during the aging processing [5]. At the beginning of the aging (from 1 to 4 h), the pre-β′′ contained Al atoms. During the transformation from pre-β′′ to β′′, Al atoms were replaced by Mg and Si, which leads to the length of precipitates decreasing after aging for 8 h. The β′′ phase grew by consuming the pre-β′′ phase, which can explain a gradual decrease in the volume fraction of the needle-shape precipitates, since some pre-β′′ phases are dissolved to provide solute atoms for the growth of β′′ phase.

## 5. Conclusions

Two aging strength peaks exist in the aging curve of wrought Al-12.7Si-0.7 Mg alloy and the strength value of the second peak is slightly higher than the first peak. The variation of mechanical properties is attributed to the combining effect of Si particles, the grain boundary, and the character of precipitates. The predominant precipitates under isothermal aging at 180 °C are pre-β′′ and β′′. A very small amount of B’ phase coexists in the matrix. As the aging time increases from 1 to 3 h, the strength increases continually, produced by the pre-β′′ growth. When pre-β′′ proceeds to a larger size, the strength decreases at 4 h. The existence of two different phases explains the shape of the aging curve: between 3 h and 8 h of aging the pre-β′′ transforms into β′′. The strength increases at 8 h as β′′ has a better hardening effect than pre-β′′. The strength of the alloy aged 12 h is decreased by the formation of a much larger β′′ precipitate.

## Data Availability

Relevant data are available from the corresponding author.
